# Localization Microscopy of Actin Cytoskeleton in Human Platelets

**DOI:** 10.3390/ijms19041150

**Published:** 2018-04-11

**Authors:** Sandra Mayr, Fabian Hauser, Anja Peterbauer, Andreas Tauscher, Christoph Naderer, Markus Axmann, Birgit Plochberger, Jaroslaw Jacak

**Affiliations:** 1 School of Medical Engineering and Applied Social Sciences, University of Applied Sciences Upper Austria, Garnisonstr. 21, 4020 Linz, Austria; fabian.hauser@fh-linz.at (F.H.); andreas.tauscher@fh-linz.at (A.T.); christoph.naderer@students.fh-linz.at (C.N.); birgit.plochberger@fh-linz.at (B.P.); jaroslaw.jacak@fh-linz.at (J.J.); 2 Red Cross Blood Transfusion Service for Upper Austria, Krankenhausstr. 7, 4017 Linz, Austria; anja.peterbauer@o.roteskreuz.at; 3 Center for Pathobiochemistry and Genetics, Institute of Medical Chemistry and Pathobiochemistry, Medical University of Vienna, 1090 Vienna, Austria; markus.axmann@meduniwien.ac.at

**Keywords:** actin cytoskeleton, Alexa Fluor 488, drift correction, dSTORM, localization microscopy, rhodamine, photo-switching, platelet shape change

## Abstract

Here, we measure the actin cytoskeleton arrangement of different morphological states of human platelets using a new protocol for photo-switching of rhodamine class fluorophores. A new medium composition was established for imaging the cytoskeleton using Alexa Fluor 488 conjugated to phalloidin. Morphological states of platelets bound to a glass substrate are visualized and quantified by two-dimensional localization microscopy at nanoscopic resolution. Marker-less drift correction yields localization of individual Alexa 488 conjugated to phalloidin with a positional accuracy of 12 nm.

## 1. Introduction

Platelets are the smallest blood cells in circulation. Upon activation—usually triggered by binding of platelets to the extracellular matrix of damaged vessel walls—reorganization of the actin cytoskeleton takes place, introducing platelet shape change. The actin cytoskeleton of platelets is described in literature as a three-dimensional, sparse, and fibrous network without apparently long actin fibers. Upon cell attachment to a surface (round-shaped cells), the actin filaments are depolymerized into shorter filaments. During spreading, new filaments are assembled—the amount of F-Actin in the activated state, however, is low (only approximately two-thirds in comparison to the resting state) [1]. Thus, characterization of the actin cytoskeleton ultrastructure within these 2–5 µm-sized cells proves arduous. In contrast to conventional fluorescence microscopy and the inherent resolution limit [2,3], localization microscopy provides insight into the distribution of specific proteins at the nanoscale and creates snapshots of cellular dynamics within single cells [4,5,6,7,8,9,10]. Thus, application of the super-resolution technique dSTORM (direct stochastic optical reconstruction microscopy) is the method of choice for these small cells [11]. The most common used fluorophore for dSTORM is the cyanine Alexa Fluor 647. The number of protocols for the application of rhodamine class fluorophores, however, is limited [12,13,14,15] and localization microscopy with dyes like Alexa Fluor 488 remains a constant challenge and relies on high-power lasers. For illumination, we used a low cost UV laser, which allowed for fast image acquisition. Fast illumination can reduce the drift, hence less correction is required.

Here, we apply dSTORM in order to display the actin cytoskeleton of platelet shape changes for the first time. For imaging we use the cyanine derivative Alexa Fluor 647 and the rhodamine class dye Alexa Fluor 488. We introduce an additional protocol for photo-switching of rhodamine derivatives like Alexa Fluor 488 in order to have an additional color channel available for labeling. The super-resolved images of platelet cytoskeleton allow quantitative characterization of the actin network, such as the width of the filopodia and of the actin accumulated at the periphery of spread platelets. Quantitatively comparable results from both color channels were obtained.

## 2. Results

### 2.1. Platelet Attachment Protocol

**Figure 1:**
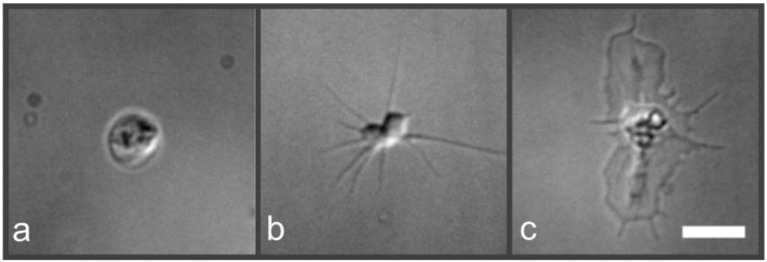
DIC images of human platelets at three different morphological states upon platelet shape change (cell culture medium on plain glass support). Upon glass contact, platelets adopt a round shape (**a**) that facilitates cell adhesion. Subsequently, filopodia form a spindle-like morphology (**b**). Lamellipodia fill the area between individual filopodia, and platelets spread further forming a so-called “fried-egg” morphology (**c**) [1,16]. Scale bar 5 µm.

To observe morphological states upon platelet shape change, we incubated the cells in cell culture medium on plain glass slides as described in literature [16,17,18,19]. Figure 1 represents typical, distinguishable platelet morphological states, visualized using differential interference contrast (DIC) microscopy. The three described states correspond to typical morphological states of platelet activation [16]. Platelet activation is a multi-factorial process that involves shape change, cell adhesion, degranulation, and ultimately clot formation [20]. The (at least partial) activation of the platelets observed here was confirmed by staining for the standard activation marker CD62p (see Supplementary Figure S1). Here we talk, however, about morphological states of platelet shape change rather than platelet activation states since the complex and multifactorial process of platelet activation was not investigated further.

Upon incubation of cells on a glass substrate, discoid platelets adopt a round shape (Figure 1a) facilitating cell adhesion, analogous to the rolling mechanism of leukocytes on activated endothelial cells of blood vessels [1]. Subsequently, highly dynamic and actin-rich cell protrusions (filopodia) serve as sensors, probing the platelet’s environment [21] and forming a spindle-like morphology (Figure 1b). Upon occurrence of lamellipodia that fill the intermediate area between individual filopodia for cell motility, cells spread further (adopting a “fried-egg” shape; Figure 1c).

### 2.2. Nanoscopic Localization of the Actin Cytoskeleton in Platelets

**Figure 2:**
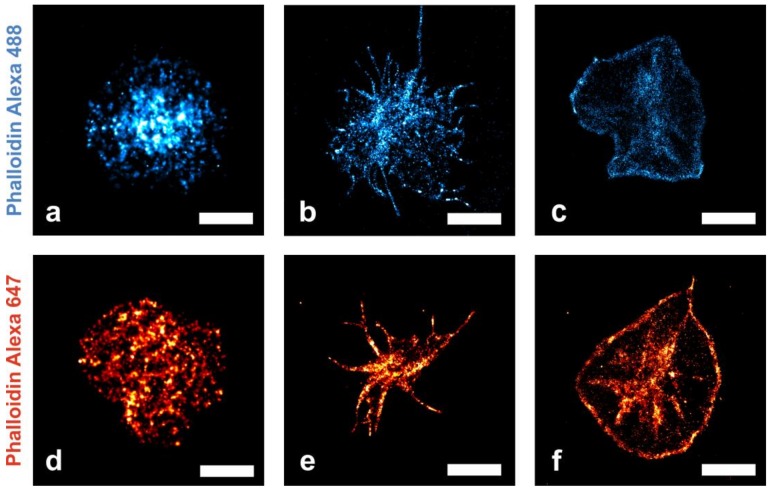
Reconstructed fluorescence microscopy images (dSTORM) of human platelets at three different morphological states (cell culture medium on plain glass support), stained with phalloidin Alexa Fluor 488 (top row) or phalloidin Alexa Fluor 647 (bottom row). F-actin molecules bound by fluorescently labeled phalloidin are represented by the red and blue colored localizations within the cells, respectively. Brighter areas are caused by a higher density of localized fluorophores. As platelets start to adhere to the glass support, an early, round-shaped appearance (**a**,**d**) transforms into a spindle-like morphology (**b**,**e**) and subsequently yields a “fried-egg” shape (**c**,**f**). The central granulomere and accumulated actin in the peripheral cell region in the ‘fried-egg’ morphology is clearly visible (Figure 2c,f) [1]. Scale bar for (**a**,**d**) 1 µm and for (**b**,**c**,**e**,**f**) 3 µm.

In this study, we present a new protocol (see Section 4) for photo-switching of the rhodamine derivative Alexa Fluor 488 (Figure 2a–c). We visualized the actin cytoskeleton of platelets that undergo morphological shape changes at the single-cell level using nanoscopic localization of fluorescently labeled phalloidins. The three exemplarily shown states correspond to typical morphological states of platelet shape change [16]. Figure 2a–c show similar morphological states of platelets labeled with Alexa Fluor 488 phallodin (additional images in Supplementary Figure S2). Figure 2a shows the cytoskeleton of a round-shaped platelet in an early state of adhesion. Figure 2b depicts a platelet in a spindle-like morphology with filopodia and Figure 2c displays a ‘fried-egg’-shaped cell with actin accumulated in the central granulomere and at the periphery of the cell.

The areas with higher local phalloidin densities in the peripheral region are of unknown origin (fluorophore induced artifact or biological origin). However, they are frequently observed mainly in “fried-egg” shaped platelets. For imaging of the rhodamine derivative Alexa Fluor 488, a new, optimized protocol was used. To ensure efficient blinking of the rhodamine dye Alexa 488 a special medium composition was applied: photo-switching is achieved with the reducing agent tris(2-carboxyethyl)phosphine (TCEP), the oxidizing agent methylviologen (MV) and β-mercaptoethylamine in a 80% glycerine solution containing 5% glucose. In this environment, the fluorophore is excited at 488 nm for 20 ms at a frame rate of 25 images/s. The fluorophore in the triplet state is reduced by TCEP and is brought back to the singlet ground state by interaction with MV and illumination with a 405 nm laser for 20 ms using a low laser power (intensity = 100 W/cm^2^). The dSTORM images were reconstructed from 15,000 frames. Alexa Fluor 488-labeled phalloidin molecules correspond to the blue colored localizations within the cells (Figure 2a–c); brighter areas correspond to a higher density of localized fluorophores. On average, we detected 740 signals/µm^2^ within a cell (*n* = 3 cells).

For verification, the platelets’ cytoskeleton was labeled with a second fluorophore conjugated to phalloidin (Figure 2d–f). The three described states correspond to typical morphological states of platelets [16]. Image acquisition was performed in a medium containing beta-mercaptoethylamine (MEA) and glycerine. The samples were irradiated at 647 nm for 20 ms. Between acquisition of individual images, the sample was illuminated with a 405 nm laser light for 10 ms. The dSTORM images were reconstructed from 15,000 frames. The localization events determined from Alexa Fluor 647 blinking are shown in red. Individual F-actin molecules bound by phalloidin conjugated to the commonly used cyanine dye Alexa Fluor 647 were localized with a 12 nm positional accuracy after drift correction for compensation of the mechanical sample movement. On average, we detected 3500 signals/µm^2^ within a cell (*n* = 3 cells).

The blinking behavior of Alexa Fluor 488 was compared to Alexa Fluor 647: Fewer signals were detected for the Alexa Fluor 488 labeled cells (740 signals/µm^2^ as compared to 3500 signals/µm^2^ for Alexa Fluor 647). However, the blinking characteristics are similar: Alexa Fluor 488 showed on average 9 ± 2 blinking events per 15,000 frames (SD = 0.1; *n* = 45 analyzed image sequences) and Alexa Fluor 647 showed 10 ± 3 blinking events (SD = 0.1; *n* = 45 analyzed image sequences).

**Figure 3:**
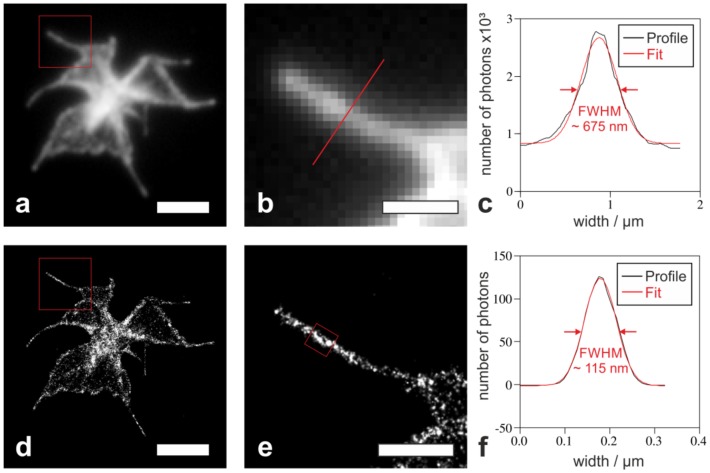
Comparison of a conventional fluorescence and a super-resolution image of the actin cytoskeleton of a platelet with a spindle-like morphology labeled with Phalloidin Alexa 647 (**a**,**d**). Close-up of the region of interest from the image in (**a**,**d**) shows a single filopodium (**b**,**e**). The intensity profile of the filopodium cross section from the boxed region (**b**,**e**) has been fitted with a Gaussian function (**c**,**f**). From the fit a FWHM of 675 nm (**c**) or of 115 nm (**f**) has been determined for the filopodium width. Scale bar for (**a**,**d**) 3 µm and for (**b**,**e**) 1 µm.

In contrast to conventional fluorescence microscopy, localization microscopy allows quantitative visualization of diffraction limited actin cytoskeleton substructures. The width of the peripheral actin network (i.e., the actin network at the edge of the ‘fried-egg’ shape as seen in Figure 2c,f) varies from 140 nm to 565 nm for Phalloidin Alexa 488 and from 200 nm to 580 nm for Phalloidin Alexa 647, respectively. Likewise, the width of filopodia was determined: Figure 3a shows the image of the actin cytoskeleton recorded with diffraction limited fluorescence microscopy; whereas Figure 3d depicts the cytoskeleton of the same cell reconstructed by localization microscopy. In Figure 3b, the filopodium from the boxed region in 3a is depicted (likewise the same filopodium at nanoscale resolution, however, seen in Figure 3e from the boxed region in Figure 3d). The width of a single filopodium (Figure 3b,e) was quantified by fitting the cross section profile with a Gaussian function. Figure 3c shows the profile of a filopodium whose width was determined (in the diffraction limited image a FWHM = 675 nm; for Phalloidin Alexa 647).

The width of the filopodium was determined from the reconstructed dSTORM image (Figure 3f): A FWHM of 115 ± 9 nm was measured for Phalloidin Alexa 647 and 106 ± 3 nm for Phalloidin Alexa 488, respectively.

Regardless of the used label, the same morphological characteristics (width of filopodia and peripheral actin network) at the same resolution (12 nm for both fluorophores) have been determined.

## 3. Discussion and Conclusions

In summary, we showed that our optimized protocol allows for recording qualitatively comparable images with Alexa Fluor 488 (compared to commonly used Alexa Fluor 647) labeled actin filaments. We showed for the first time the actin cytoskeleton of three different platelet morphological states resolved at a resolution beyond the diffraction limit. In contrast to the dSTORM images, the conventional fluorescence microscopy images of filopodia appeared as diffraction limited structures: Only the dSTORM images allowed us to determine the size distribution of platelets’ filopodia width. A detailed quantification revealed an average filopodium width of 115 ± 9 nm (for Phalloidin Alexa 647) and 106 ± 3 nm (for Phalloidin Alexa 488), respectively. Apart from a report exclusively showing a filopodium width of a neuroblastoma cell [22] this is the first quantitative description of the width of filopodia and the very first on filopodia in human platelets.

Photo-switching of the rhodamine dye Alexa Fluor 488 is achieved in a special medium composed of the reducing agent TCEP, the oxidizing agent MV and glycerine as well as an optimized illumination protocol. Using this protocol allows for implementation of standard (i.e., low cost) diode UV laser sources in contrast to usually applied high-end laser sources. In addition, the applied marker-less drift correction enables acquisition of a sequence with a higher number of frames, thereby compensating the reduced number of signals/µm^2^ in the blue channel. The applicability of the new medium for rhodamine class dyes is shown with Alexa Fluor 555 (Supplementary Video S1).

The medium applied here can be mixed with additives like oxygen scavengers to improve photo-switching properties of the dye [15]. A medium with higher refractive index (glycerine, *n* = 1.45) [23] compared to aqueous solutions allows us to detect more photons from a single fluorophore and a more precise position determination. Such high positional accuracy as obtained by our protocol allows for precise monitoring of distinct morphological changes. For filopodia width determination, our obtained 12 nm resolution is completely sufficient. Images of filopodia reconstructed with a lower resolution verify that even a resolution of ~20 nm is satisfactory (Supplementary Figure S3).

The use of different fluorophores for dSTORM, as demonstrated by qualitative comparison of the platelet actin cytoskeleton stained with phalloidin Alexa Fluor 647 as well as phalloidin Alexa Fluor 488, offers flexibility for multi-color imaging in the future and can be applied for any desired cell (Supplementary Figure S4).

## 4. Materials and Methods

### 4.1. Human Platelet Concentrates

Single donor platelet concentrates were provided by the Red Cross Blood Transfusion Service (Linz, Upper Austria, Austria). Platelet concentrates were prepared by apheresis with an automated cell separator (Trima Accel Automated Blood Collection System, TerumoBCT, Lakewood, CA, USA) during routine thrombophoresy: The platelets were separated from whole blood by centrifugation and diluted in 35% plasma, 65% platelet additive solution SSP+ (Macopharma, Mouvaux, France), and ACD-A anticoagulant (Haemonetics^®^ anticoagulant citrate dextrose solution, Haemonetics^®^, Braintree, MA, USA) during transfer into Trima Accel storage bags. Two milliliters of the platelet concentrate (typically containing 1 × 10^6^ platelets/µL) were transferred into a new storage bag and immediately transported to the laboratory. Transportation within a polystyrene box should minimize temperature variations. Platelets were used for experiments within 24 h after preparation and meanwhile stored under constant agitation in a climatic chamber that was tempered to 22 °C.

### 4.2. Statement on the Use of Human Platelet Concentrate Samples

All human blood samples were collected during routine thrombophoresy in accordance with the strict policies of the Red Cross Transfusion Service, Linz. All blood donors signed their informed consents that residual blood material can be used for research and development purposes. All experimental protocols were approved by and carried out in collaboration with the Red Cross Blood Transfusion Service, Linz.

### 4.3. Platelet Spreading, CD62p and Actin Staining

Platelets were diluted to a final concentration of 2 × 10^4^ cells/mL in cell culture medium (DMEM, Sigma-Aldrich, Vienna, Austria), pipetted onto a glass slide and incubated for 15 min. Non-adhered cells were washed away with PBS (phosphate-buffered saline). For DIC microscopy, cells were fixed using 4% paraformaldehyde in distilled water and imaged. The actin cytoskeleton was visualized using Alexa Fluor 647 phalloidin and Alexa Fluor 488 phalloidin (Cell Signaling Technology, Leiden, The Netherlands), respectively. Platelet actin staining was performed in Cytoskeleton buffer with sucrose (CBS) containing 10 mM MES pH 6.1, 138 mM KCl, 3 mM MgCl_2_, 2 mM EGTA, and 0.32 M sucrose according to a protocol of Louise Cramer (MRC Laboratory for Molecular Cell Biology, UCL, London, UK) [24]. Briefly, platelets were fixed using 4% paraformaldehyde in CBS for 20 min at room temperature, permeabilized in 0.5% Triton X-100 with CBS, blocked in 10% albumin from chicken egg white (Sigma-Aldrich, Vienna, Austria) and stained for 20 min with 66 nM fluorophore conjugated to phalloidin. For CD62p labeling, fixed cells were stained with 1 µg/mL anti-CD62p antibody marked with Alexa 647 (BioLegend, San Diego, CA, USA) for 10 min.

### 4.4. Fluorescence Microscope

Images were acquired using a modified Olympus IX81 inverted epifluorescence microscope with an oil-immersion objective (UApo N 100x/1.49 NA, Olympus, Vienna, Austria). The sample was positioned with nanometer precision on a XYZ piezo stage (P-733.3DD, Physical Instruments) on top of a mechanical stage with a range of 1 × 1 cm adjusted by precision screws (TAO, JPK Instruments, Berlin, Germany). A tube-lens with an additional magnification of 1.6 was used to achieve a final imaging magnification of 160 (corresponding to a pixel size of 100 nm). Platelets were illuminated with a 642 nm laser light from a diode laser (Omicron-laserage Laserprodukte GmbH, Phoxx 642, Rodgau-Dudenhofen, Germany), a 488 nm laser light from a solid-state laser (diode-pumped, Toptica Photonics, Graefelfing, Germany), and a 405 nm laser light from a diode laser (Insaneware, Gladbeck, Germany). The signal was detected using an Andor iXonEM+ 897 (back-illuminated) EMCCD camera (16 μm pixel size). The following filter sets were used: dichroic filter (ZT405/488/561/640rpc, Chroma, Olching, Germany), emission filter (446/523/600/677 nm BrightLine quad-band band-pass filter, Semrock, Rochester, NY, USA), and an additional emission filter (HQ 700/75 M, NC209774, Chroma Technology GmbH, Olching, Germany).

### 4.5. Imaging Protocols

Single-molecule photo-switching of the cyanine dye Alexa Fluor 647 was performed in a 50 mM β-mercaptoethylamine in 80% glycerine containing medium (refractive index 1.45) as described by Huang and co-workers [8] and applied to the cells immediately prior to fluorescence microscopy measurements. The signal was acquired for 20 ms at 25 images/s at 1.2 kW/cm^2^ excitation intensity (647 nm). During the readout, laser light from the 405 nm laser (10 ms illumination at 100 W/cm^2^) was used to recover the singlet ground state. For image reconstruction, a sequence of 15,000 images was recorded.

Single-molecule photo-switching of the rhodamine dye Alexa Fluor 488 was performed in a medium composed of 2 mM tris(2-carboxyethyl)phosphine, 2 mM methylviologen, 50 mM β-mercaptoethylamine in 80% glycerine with 5% glucose in distilled water (one example of a single molecule can be found in Supplementary Figure S5). The signal was acquired during 20 ms with 20 ms delay at 3.3 kW/cm^2^ excitation intensity. Between acquisition of individual images, laser light from the 405 nm laser (10 ms illumination at 100 W/cm^2^) was used to recover the singlet ground state. For image reconstruction, a sequence of 15,000 frames was recorded. The illumination protocols were performed with a custom-written acquisition software.

### 4.6. Image Analysis

dSTORM image series were processed and analyzed using custom-written software (‘STORM Tools’) in Qt/C++ and is freely available at (GITHUB https://github.com/CURTLab/STORMTools). The high-performance non-maximum suppression algorithm is adapted (with permission) from rapidSTORM [25] and used to approximate the positions of single molecules in each frame [26,27]. Each fit is performed in a window surrounding the proposed position. The point spread function (PSF) is fitted using the symmetrical two-dimensional Gaussian error function model [28] and successively approximated by the trust-region Powell dogleg algorithm [29]. The resulting parameters (x-position, y-position, integrated signal intensity, background intensity, signal width) are further used to calculate the positional accuracy [30] for the super-resolution reconstruction. One of the parameters, which is additionally needed for estimation of the positional accuracy is the background variability. Each molecule is rendered at its determined (x,y)-position using a symmetrical Gaussian intensity distribution with the calculated positional accuracy as width parameters (Supplementary Figure S6). To correct for the linear drift of the stage, each localization set has been corrected before image reconstruction (see drift correction) (Supplementary Figure S7a,c). In order to determine the lower boundary for the localization precision, the mechanical stability of the whole instrument has been determined via imaging of TetraSpeck™ beads (T-7284, 0.1 µm, Molecular Probes) over 10,000 frames within 7 min (20 ms illumination time, excitation at 647 nm). The determined radial displacement relative to the averaged signal position is 3 nm (Supplementary Figure S7b,d), which sets the technical lower limit for the positional accuracy for drift-corrected localized images. 

### 4.7. Drift Correction

#### 4.7.1. Drift Calculation Method

The drift correction method has been applied according to Han et al. [31]. The concept of drift correction is similar to cross correlation analysis and is based on the Parzen-window density estimation [32,33]. 

The method uses the determined molecule positions to judge the similarity of two signal distributions. This is conceptually straightforward and easier to operate in comparison to the cross-correlation analysis.

Assuming there are two position sets SA and SB ($p_{i}\in S_{B},j=1,2,\ldots N$ and $p_{j}\in S_{B},j=1,2,\ldots M$), the cost function CF(T) of drift correction T is given by

$$\mathrm{CF}\left(T\right)=\sum_{j}^{M}\sum_{i}^{N}\frac{1}{2{\pi \sigma }^{2}}\exp\left(-||T\left(p_{j}\right)-p_{i}||{}^{2}/2\sigma^{2}\right)$$

where T(·) is a transformation function (translation and/or rotation of positions), $S_{B}$ is movable, and $S_{A}$ is fixed (basis set of positions). The goal is to find an T(·) that maximizes the CF. The simplest transformation is linear (only translation), i.e., T(p)=p+d, where $d={\left(\alpha ,\beta \right)}^{T}$ is shift vector, α is the drift compensation of the *x*-axis and β is the drift compensation of the *y*-axis.

#### 4.7.2. Drift Correction Algorithm

First, the reference position set (basis set of positions) S_basis_ is initialized. Hence, the positions within frames are combined into groups S(i). Equivalent time intervals or an equivalent number of points per group arranges the groups. By comparison if the position set S(i) to the S_basis_, the sample drift is estimated. Hence, the drift of S(i) is compensated and the positions of S(i) is merged with S_basis_. A quasi-newton algorithm is used to find maximum of function CF(T). Repeat the selection and drift compensation process until all the point subsets S(i) are corrected. The last reference position set in S_basis_ is the referenced corrected data. 

### 4.8. Grouping Process

The data of individual frames are grouped in two ways:

First, the same length of time interval (the same number of frames in the frame-group). This may result in frame-groups with a small number of positions (e.g., 1 or 2 points). In this case, the translation vector d can be very long. In order to avoid that, the drift length will be limited by max-limit-drift, calculated automatically from the accuracy (sigma) of the Gaussian fitting of individual signals.

Second, the same number of points (positions) in each frame-group. In this case grouping with a fixed number of points introduce variable illumination time intervals.

### 4.9. Smoothing

Smoothing of the time dependent drift of points is carried out by two methods:

- Fitting ‘Smoothing-Spline’ with parameter 10^−4^ only in the discrete time window (sliding along the time-axis).
- Fitting method ‘Smoothing-Spline’ with parameter 10^−6^ for the entire time line.

## Supplementary Materials

Supplementary materials can be found at http://www.mdpi.com/1422-0067/19/4/1150/s1.

## Abbreviations

dSTORM	Direct stochastic optical reconstruction microscopy
FWHM	Full width at half maximum
DIC	Differential interference contrast
TCEP	Tris(2-carboxyethyl)phosphine
MEA	β-mercaptoethylamine
MV	Methylviologen
CBS	Cytoskeleton buffer with sucrose
ACD-A	Acid citrate dextrose and adenosine
CF	Cost function
PSF	Point spread function
