# Supplementary material for: Localization Microscopy of Actin Cytoskeleton in Human Platelets
Source: Int J Mol Sci. 2018 Apr 11;19(4):1150. doi: 10.3390/ijms19041150 (PMC5979344; doi:10.3390/ijms19041150)
Supplement: Supplementary file 1 [file ijms-19-01150-s001.zip › Supplementary final.pdf]

International Journal of *Molecular Sciences*

MDPI

Supplementary

Localization Microscopy of Actin Cytoskeleton in Human Platelets

Sandra Mayr [1,*], Fabian Hauser [1], Anja Peterbauer [2], Andreas Tauscher [1], Christoph Naderer [1], Markus Axmann [3], Birgit Plochberger [1], Jaroslaw Jacak [1]

[1] School of Medical Engineering and Applied Social Sciences, University of Applied Sciences Upper Austria, Garnisonstr. 21, 4020 Linz, Austria; fabian.hauser@fh-linz.at (F.H.); andreas.tauscher@fh-linz.at (A.T.); christoph.naderer@students.fh-linz.at (C.N.); birgit.plochberger@fh-linz.at (B.P.); jaroslaw.jacak@fh-linz.at (J.J.)

[2] Red Cross Blood Transfusion Service for Upper Austria, Krankenhausstr. 7, 4017 Linz, Austria; anja.peterbauer@o.roteskreuz.at

[3] Center for Pathobiochemistry and Genetics, Institute of Medical Chemistry and Pathobiochemistry, Medical University of Vienna, 1090 Vienna, Austria; markus.axmann@meduniwien.ac.at

* Correspondence: sandra.mayr@fh-linz.at; Tel.: +43-50804-56017

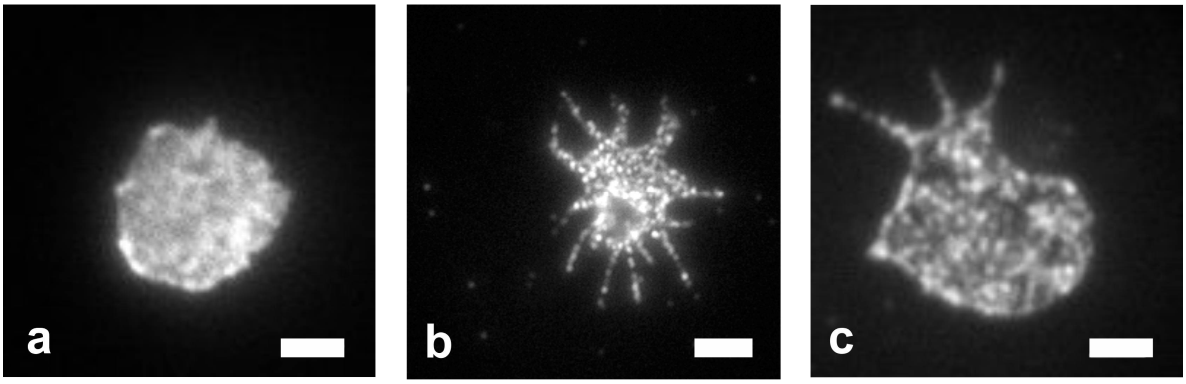

Supplementary Figure 1: Platelets of different morphology were stained with Alexa Fluor 647-conjugated antibodies against the standard activation marker CD62p. The round-shaped state of a platelet (a) at the beginning of adhesion and the spread cells with filopodia (b) as well as lamellipodia (c) show CD62p expression. Scale bar 2 µm for (a) and 4 µm for (b, c).

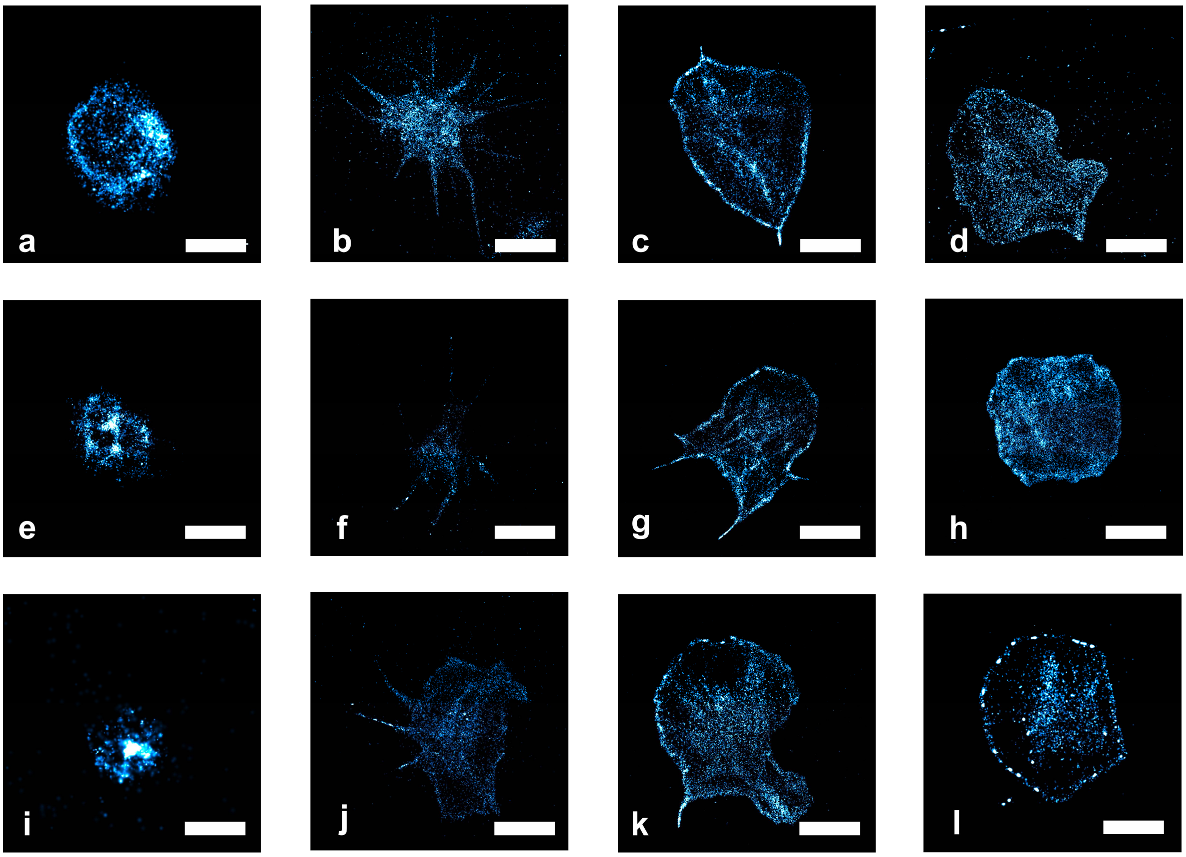

Supplementary Figure 2: Reconstructed fluorescence microscopy images (dSTORM) of human platelets with different morphological states of shape change (cells in cell culture medium on plain glass support, fixed with paraformaldehyde) and stained with phalloidin Alexa Fluor 488. F-actin molecules bound by fluorescently labeled phalloidin are represented by blue colored localizations within the cells. Brighter areas are caused by a higher density of localized fluorophores. Each image constitutes a different cell. Scale bar for (a, e, f) 1 µm and for (b-d, f-h, j-l) 3 µm.

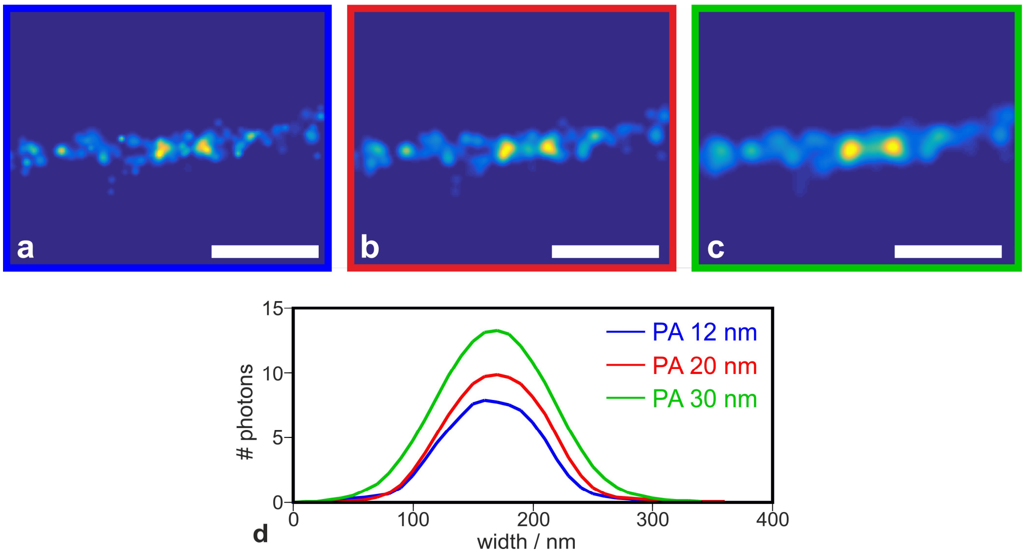

Supplementary Figure 3: Reconstructed images of the same platelet filopodium with different resolutions of 12 nm (a), 20 nm (b), and 30 nm (c). The discontinuous structure seen with a 12 nm resolution in (a) changes into a continuous filament if a lower resolution of 30 nm (c) is used. Scale bar 500 nm (a, b, c).

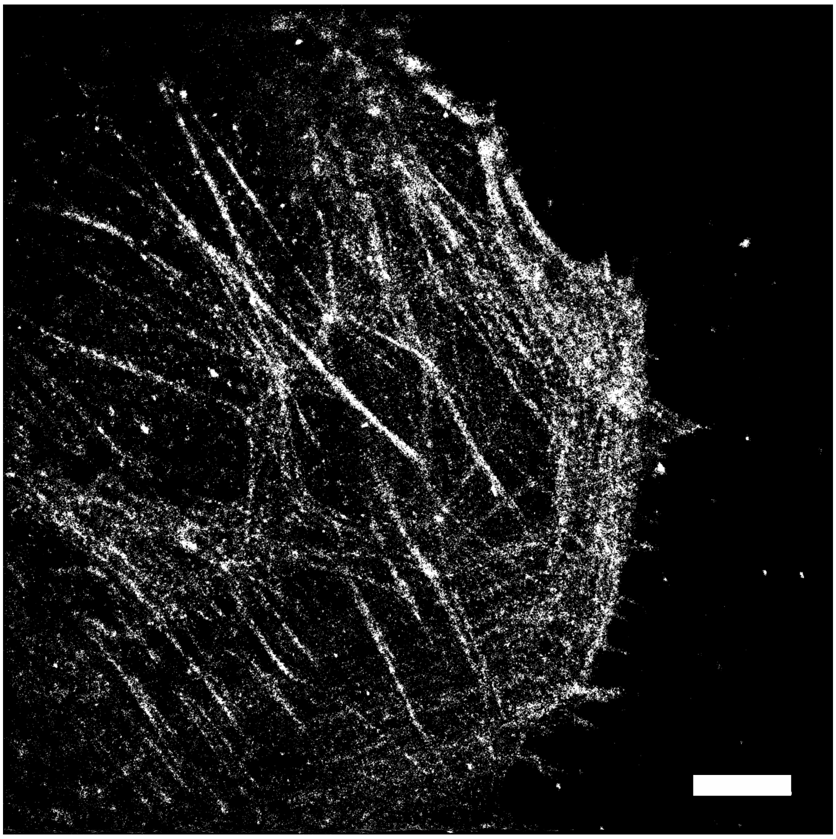

Supplementary Figure 4: Reconstructed fluorescence microscopy image (dSTORM) of an endothelial cell (HUVEC) stained with phalloidin Alexa Fluor 647. Fluorescently labeled F-actin molecules are represented by white marked localizations within the cell. Scale bar 5 µm.

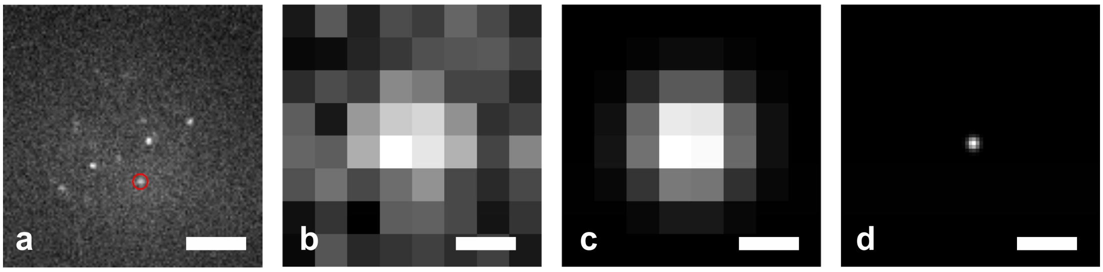

Supplementary Figure 5: Fluorescence signal of a representative frame taken from a dSTORM sequence (Phalloidin Alexa 647) (a). One exemplary signal (red circle in (a)) is magnified as shown in (b). The signal has an intensity of 642 photons (intensity over the whole signal without background**)**, a background of 126 photons (variability of 3 photons) and a FWHM of 300 nm. The signal from (b) is fitted and reconstructed with its original width (c). For the reconstruction of the super-resolution image, a PA of 1 nm is used (d). Scale bar 3 µm (a), 250 nm (b,c,d).

Positional Accuracy

The position of the signal of an individual molecule is fitted by a Gaussian function. The width (σ) of the reconstructed intensity distribution of a single molecule is calculated from its positional accuracy (PA). The formula for the calculation of the positional accuracy is given by Mortenson et al [24] and depends on the number of photons (N), sigma of the estimated point spread function (σ), the pixel size (a) and the expected number of background photons per pixel (b^2). The value b is the standard deviation of the local background per pixel. In order to determine the variance of the background b, an average variance of pixel intensities along a square boundary line of the fitting window has been determined. This methodology is thereby free from assumptions and independent from the PSF model.

$$\mu(N, \sigma, a, b) = \frac{\sigma^2 + a^2/12}{N}\left(\frac{16}{9} + \frac{8\pi(\sigma^2 + a^2/12)b^2}{Na^2}\right)$$

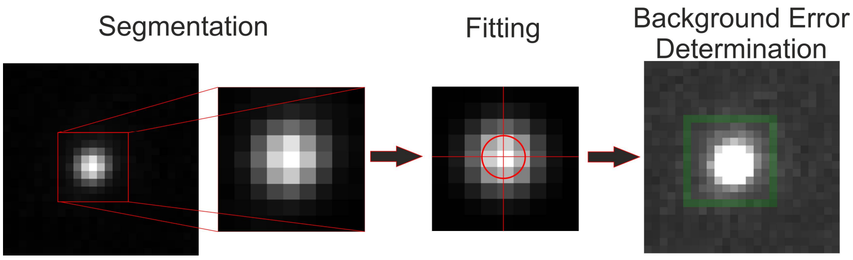

Supplementary Figure 6: Signal detection. The Non-Maximum-Suppression algorithm segments a single frame into several regions of interests (ROIs). Each ROI contains a single signal and is used for Gaussian fitting. After the determination of the PSF-parameters (x-position, y-position, integrated signal intensity, signal width, background intensity), the standard deviation of the local background is calculated.

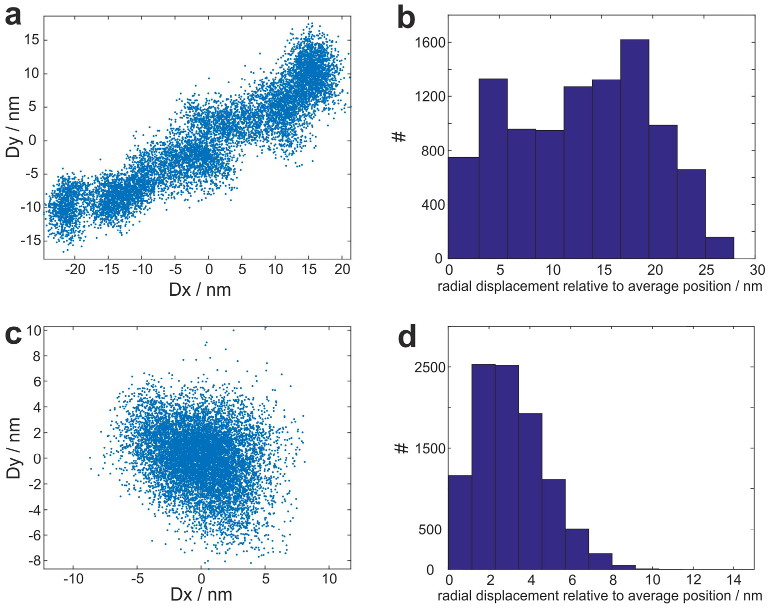

Supplementary Figure 7: Determination of the lower technical limit for the positional accuracy. Individual Tetraspeck™ beads are observed over 10,000 frames (20 ms illumination time, excitation at 647 nm, total measurement time 7 minutes). (a) Individual position of a single bead: Clearly observable is a linear drift of the bead. (b) Histogram of the radial displacement. The calculated lower technical limit for the PA is 13 nm, which is the mean of the displacement. (c) X, Y-position of the bead (see (a)) after application of the marker-less drift correction. (d) Histogram of the corresponding distribution around the center position of the fluorophore over 10,000 frames. After applying of the drift correction, the mean radial displacement relative to the average position is reduced to 3 nm.

Supplementary Video 1: Blinking of the rhodamine fluorophore Alexa Fluor 555 conjugated to a anti-CD59 antibody bound on platelets. Photo-switching is achieved with the here presented protocol.
